# Potential Advantages of a Well-balanced Nutrition Regimen for People Living with Human Immunodeficiency Virus Type -1

**DOI:** 10.33696/aids.6.048

**Published:** 2024

**Authors:** Daniele Basta, Olga S. Latinovic, Yutaka Tagaya, Giovannino Silvestri

**Affiliations:** 1 Green Home scarl, Scientific Committee, Arcavacata di Rende (CS), Italy; 2 Department of Microbiology and Immunology, School of Medicine, University of Maryland, Baltimore, MD, 21201, USA; 3 Division of Virology, Pathogenesis, and Cancer, Institute of Human Virology, University of Maryland School of Medicine, MD, 21201, USA; 4 Department of Medicine, School of Medicine, University of Maryland, Baltimore, MD, 21201, USA; 5 Marlene and Stewart Greenebaum Comprehensive Cancer Center, University of Maryland, Baltimore, MD, 21201, USA

**Keywords:** Implications for nutritional interventions in HIV-1 care, Macronutrients, Metabolic complications in HIV-1 patients, Micronutrients in immune function, The challenges of nutritional deficiencies

## Abstract

This review underscores the important role of nutrition in enhancing the management of Human Immunodeficiency Virus type 1 (HIV-1). Highlighting the efficacy of dietary interventions, including, the importance of omega-3 fatty acids, vitamins D and B-12, and the Mediterranean diet, we delineate how these beneficial nutritional strategies can improve the effectiveness of combined antiretroviral therapy (cART), mitigate its side effects, and ameliorate metabolic disorders in people living with HIV-1 (PLWH). Our review advocates for the integration and implementation of personalized nutritional assessments into the care plan for PLWH, proposing actionable strategies for healthcare providers in HIV-1 field. Summarizing the current standing of the relevance of the nutritional and well-planned diet recommended for the PLWH and emphasizing on the future research directions, this review establishes a foundation for nutrition as a cornerstone in comprehensive HIV-1 management. Our review aims to improve patients’ health outcomes and overall quality of life for PLWH.

## Introduction

Effective management of HIV-1 remains a complex, multifaceted challenge in the global public health landscape. The advantage of cART has marked significant progress in HIV-1 treatment, reducing mortality and improving quality of life for PLWH [1,2]. Despite these advancements, patients continue to face numerous health challenges exacerbated by the virus and antiretroviral treatment. These include, but are not limited to, opportunistic infections, cardiovascular diseases, renal dysfunction, neurocognitive disorders, and a heightened risk for certain malignancies. The direct impact of HIV-1 on vascular inflammation and endothelial dysfunction, alongside cART side effects such as dyslipidemia and insulin resistance, further complicates the clinical picture for PLWH, accelerating atherosclerosis and altering lipid metabolism [3–5].

Moreover, HIV-1 associated nephropathy (HIVAN) [6,7] and HIV-associated neurocognitive disorders [8] present significant concerns, affecting patient health and quality of life. The persistence of stigma surrounding HIV-1, alongside the metabolic complications induced by cART, such as weight gain and its associated risks, underscores the complexity of managing HIV-1 suppressing virological control. The role of nutrition emerges as an important element in this context, offering a modifiable factor that can influence the course of the disease and the well-being of those affected [9].

Nutritional interventions, tailored to address the unique needs of individuals undergoing cART, have shown potential in mitigating some of these challenges, emphasizing the importance of a balanced diet rich in essential nutrients [10]. Research highlights the role of specific dietary components, such as omega-3 fatty acids and micronutrients, in supporting immune function, reducing inflammation, and managing metabolic disturbances [11]. Recent clinical trials have explored various nutritional interventions in PLWH undergoing cART, focusing on their impact on patients’ health outcomes, including metabolic parameters and nutritional status [12–16].

This review aims to elucidate the multifaceted interactions between nutrition and HIV-1 management, exploring the impact of dietary strategies on improving HIV-1 treatment outcomes. By examining the current evidence on nutritional interventions, including the use of supplements and the adoption of specific dietary patterns, we seek to provide comprehensive insights into optimizing the care of PLWH. Emphasizing the integration of nutritional strategies into the broader HIV-1 management approach, we underscore the potential of nutrition to enhance the effectiveness of cART, mitigate its side effects, and contribute to the overall quality of life for PLWH.

In conducting this review, a systematic approach was adopted to explore the intersection of nutrition with HIV-1 management. Literature searches were performed using key terms such as: nutrition and HIV-1, nutrition and cART, and nutrition and AIDS. These searches spanned several scientific databases to ensure comprehensive coverage of all available and relevant studies to date. The inclusion criteria focused on research that explicitly examined nutritional interventions and their beneficial impacts on HIV-1 outcomes. Exclusion criteria were applied to filter out any studies that did not meet the quality and relevance of our standards. This meticulous process enabled the synthesis of data from a broad spectrum of scientific works, providing a robust foundation for our analysis.

### Comorbidities and metabolic complications in PLWH

Maintaining a nutritious diet and healthy lifestyle is important for anyone, whether they live with HIV/AIDS or not. HIV-1 significantly compromises the immune system, presenting it as a severe and fatal disease, if not on cART. In addition to compromised immunity, HIV-1 patients face risks such as opportunistic infections and oxidative stress [9]. During combined cART, PLWH may undergo body and face shape changes, bone mass loss, diarrhea, lean tissue depletion, elevated blood lipids, and other metabolic complications, each contributing to an increased risk of mortality [17].

The health challenges experienced by PLWH are compounded by a spectrum of comorbidities, including cardiovascular diseases, renal dysfunction, and neurocognitive disorders [18–20]. Cardiovascular diseases are particularly prevalent in this HIV-1 population. This heightened challenge can be attributed to a combination of factors: the direct impact of the HIV-1 virus itself, which may induce vascular inflammation and endothelial dysfunction, and the various side effects of cART. That includes altered lipid metabolism and insulin resistance, leading to accelerated atherosclerosis [21–23].

Renal dysfunction in PLWH is another area of potential concern. HIV-associated nephropathy, a direct consequence of the viral infection of renal epithelial cells, represents one of the most severe forms of renal impairment in this population [17,18]. This condition is characterized by collapsing focal segmental glomerulosclerosis and tubular injury, which can lead to chronic kidney disease and, ultimately, end-stage renal disease [6,18]. The prevalence of HIVAN varies geographically and ethnically but is higher among African Americans [7]. The pathogenesis of HIVAN involves direct viral effects, genetic predispositions (such as variants in the Apolipoprotein L1 gene), and immune-mediated mechanisms [7].

Neurocognitive disorders are also frequently observed in PLWH, ranging from mild, asymptomatic neurocognitive impairment to more severe forms like HIV-associated dementia [8,24,25]. These conditions are thought to arise from both direct viral invasion and the resultant chronic immune activation within the central nervous system. HIV-1 enters the brain early after infection, leading to a cascade of events including neuroinflammation and synaptic damage, which over time can result in significant cognitive, motor, and behavioral impairments [8]. Additionally, PLWH are at increased risk for certain malignancies. Kaposi sarcoma and non-Hodgkin lymphoma are particularly frequent, often linked to coinfections with oncogenic viruses such as human herpesvirus 8 and Epstein-Barr virus, respectively [26–28]. These cancers are thought to emerge from a complex interplay of immunosuppression, chronic inflammation, and viral oncogenesis [26]. The risk of these cancers remains elevated in PLWH despite effective cART, highlighting the need for ongoing cancer surveillance and prevention strategies in this population [27,28].

### Addressing stigma and metabolic challenges in comprehensive HIV-1 care

Stigma associated with HIV-1 and its treatment continues to be a pervasive issue, impacting mental health, quality of patients’ life, and treatment adherence. The stigma can be manifested in various forms, from societal discrimination to internalized stigma, and is a key factor to address in comprehensive HIV-1 care strategies [29–31]. Furthermore, the scenario involving potential side effects like weight gain leading to obesity and cardiometabolic disease is increasingly recognized. Obesity can exacerbate the risk of cardiovascular diseases, diabetes, and other metabolic disorders, complicating the management of HIV-1. This association between cART, particularly integrase inhibitors, and weight gain and its subsequent metabolic consequences is an area of active research. It underscores the need for regular monitoring of body weight and metabolic parameters in patients on cART, alongside interventions to manage these risks, such as dietary modifications and physical activity [32–34]. Among all these obstacles, a patient’s weight increase stands out as a controllable factor. This is needed because individuals with HIV-1 can proactively adopt improved dietary habits, recognizing that qualitative nutrition as a relevant aspect for their well-being and better disease management. Additionally, the growing risk for those with HIV-1/AIDS and condition of obesity includes an increased risk for lipodystrophy (uncontrollable deposition of fat) was introduced [17]. Specifically, recent reports discuss the presence of lipohypertrophy (gain of truncal fat) with the newer generation of cART drugs [35]. Among the first generation of antiretrovirals, thymidine analogs like stavudine (d4T) and zidovudine (AZT) were frequently associated with lipoatrophy, characterized by the loss of subcutaneous fat, particularly in the face, limbs, and buttocks [36]. Early PIs (Protease Inhibitors), such as ritonavir (RTV), indinavir (IDV), and nelfinavir (NFV), were linked to lipohypertrophy, manifesting as fat accumulation in the abdominal area, dorsocervical region (buffalo hump), and breasts. This fat redistribution often occurred in conjunction with metabolic abnormalities, including dyslipidemia and insulin resistance [17,37,38]. More recent reports have associated newer antiretrovirals, particularly Integrase Strand Transfer Inhibitors (INSTIs) like Cabotegravir (CAB), dolutegravir (DTG), raltegravir (RAL), and elvitegravir (EVG), with weight gain and lipohypertrophy [17].

The mechanism underlying the development of lipohypertrophy in the context of cART, especially with agents like CAB, is not fully elucidated but is thought to involve alterations in lipid metabolism and adipose tissue distribution [17]. Furthermore, the interaction between antiretroviral therapy and nutritional status is intricate, encompassing a potential for both direct and indirect influences on the physiological distribution of body fat and lean mass. These complex dynamics can significantly impact the overall health and well-being of individuals undergoing HIV-1 treatment.

### Integrated challenges in HIV-1 management and the crucial role of nutrition

#### Recent HIV-1 statistics and pre-exposure prophylaxis (PreP):

HIV-1 remains a formidable challenge in the global public health landscape, having resulted in the loss of 40.4 million lives within the statistical range of 32.9 to 51.3 million. As of the conclusion of 2022, an estimated 39.0 million individuals were living with HIV-1 worldwide. Additionally, in the year 2022 alone, approximately 1.3 million individuals, within the range of 1.0 to 1.7 million, contracted HIV. The global impact of HIV-related causes in 2022 manifested in the mortality of 630,000 people, with a confidence interval spanning from 480,000 to 880,000 [39]. These figures underscore the ongoing imperative for effective prevention, treatment, and a cure on a global scale. Recent advancements in antiretroviral therapy encompass pre-exposure prophylaxis (PreP) [40–42]. PreP represents a groundbreaking approach in HIV-1 prevention management. It involves regular intake of antiretroviral drugs by individuals who do not have HIV-1 but are at substantial risk of contracting the virus. This method has shown remarkable efficacy in reducing the risk of HIV-1 transmission among high-risk populations, including those with HIV-1 positive sexual partners, individuals with a history of inconsistent condom use, and certain key populations like sex workers and intravenous drug users [43].

The importance of PreP in the context of global HIV-1 prevention cannot be overstated. By effectively reducing the risk of HIV-1 transmission, PreP plays a crucial role in curtailing new infections. This is particularly vital in regions and communities where the prevalence of HIV-1 is high, and access to comprehensive healthcare services might be limited [43]. Moreover, the implementation of PreP programs goes hand in hand with increased awareness and education about HIV-1, further contributing to the broader efforts of HIV-1 prevention. The availability of PreP also underscores the shift in HIV-1 prevention strategies from solely behavior-based methods to a more integrated approach that includes biomedical interventions. Additionally, there is a growing focus on investigating the impact of proper nutrition for HIV-1 patients undergoing treatments, recognizing its potential benefits in conjunction with antiretroviral interventions and lowered side effects [44–46].

#### HIV-1, cART and benefits of proper nutrition:

Significant strides have been achieved in the diagnostics and treatment of AIDS since the discovery of HIV-1, in 1983 [47–49]. The remarkable effectiveness of cART is evident through substantial reductions in mortality, successful control of peripheral blood viral load, stopping viral transmission, and the achievement of a nearly normal quality of life for HIV-1 [50,51]. However, the cART is not curative as it does not eradicate persistent viral reservoirs that are established once the viral genome integrates into the host genome. In addition, the prolonged administration of cART introduces health risks, including diabetes, obesity [52], and cardiovascular disease (CVD) [53,54], collectively referred to as metabolic syndrome [54]. Recent attention has shifted towards the implementation of proper diets for PLWH to bolster the immune system and enhance the effectiveness of cART [9].

The treatment and management of HIV-1/AIDS remains fraught with evident challenges, each necessitating a nuanced understanding and approach. Drug toxicities, a critical issue, manifest in various forms ranging from mild to severe and can significantly impact patient adherence to antiretroviral therapy. For instance, cART-related hepatotoxicity and nephrotoxicity have been well-documented in the literature, highlighting the need for careful monitoring of liver and kidney function in patients undergoing treatment [55]. Viral resistance to cART is another formidable challenge. The high mutation rate of HIV-1 enables the virus to quickly adapt and develop resistance to drugs, especially in cases of suboptimal adherence to therapy or inadequate drug levels. This resistance can compromise the effectiveness of existing treatment regimens and necessitates for the ongoing development of new drugs and combination therapies [10,56]. The presence of HIV-1 reservoirs, where the virus remains hidden and inactive, even after sustained virological suppression with cART is a major barrier to curing the HIV-1 infection. These reservoirs can reignite infection if cART treatment is stopped. They are typically located in various tissues and cell types, including CD4^+^ T cells, lymphoid tissues, gut, and central nervous system. They present a significant hurdle in achieving a sterilizing or functional cure for HIV-1 [57]. The lifelong cost of cART is also a concern, particularly in resource-limited settings. The financial burden of continuous medication, along with the necessary monitoring and management of side effects and comorbidities, poses a challenge for patients and healthcare systems alike. This aspect underscores the need for more cost-effective treatment strategies and the importance of global efforts to improve access to affordable HIV-1 medications [58].

In addition, ensuring proper nutrition not only supports immune function but also enhances the effectiveness of cART. It contributes to an improved overall quality of life for PLWH, including a positive impact on body mass index (BMI) [10,59,60]. Once cART is established, maintaining a nutritionally sound diet remains relevant due to cART’s metabolic effects, such as obesity, insulin resistance, and dyslipidemia [61].

When continuing the theme of nutrition in the context of HIV-1 infection, it is highly recommended to delve into the multifaceted interactions between dietary components and cART. For example, nutritional deficiencies can exacerbate the immunosuppression caused by HIV-1, thereby diminishing the therapeutic efficacy of cART. On the other hand, a well-balanced diet enriched in essential nutrients can boost the immune system, thus enhancing the body’s natural defenses against opportunistic infections commonly associated with AIDS.

Furthermore, research has shown that certain micronutrients play an important role in modulating the progression of HIV-1 infection (Table 1). For instance, deficiencies in vitamins A, B6, B-12, C, D, E, and minerals like zinc and selenium have been linked to faster disease progression and increased mortality in people living with HIV [10,62,63]. These micronutrients are important for immune function; for example, Vitamin D is known to enhance the pathogen-fighting effects of monocytes and macrophages, cells that are vital in the immune response against HIV-1 [64].

In addition to micronutrients, macronutrients also play a significant role in managing HIV-1 infection. A diet balanced in proteins, carbohydrates, mono- and polyunsaturated fats has been proved to benefit maintaining of the lean body mass and preventing wasting syndrome, a condition commonly seen in advanced stages of HIV-1 infection. Protein-energy malnutrition has been identified as a present risk factor for mortality in PLWH, further underscoring the importance of adequate nutrition [65–67]. Moreover, the interplay between cART and nutrition extends beyond mere immune function. cART, especially PIs, has been associated with metabolic complications such as lipodystrophy, hyperlipidemia, and insulin resistance [68,69]. These cART-induced metabolic changes can be mitigated through dietary modifications and physical activity, emphasizing the need for comprehensive nutritional management in PLWH.

In the context of managing HIV-1, it is recommended that PLWH adopt a well-considered, balanced, and proactive long-term dietary strategy. This dietary strategy plays an important role in maintaining optimal nutritional status and leveraging the benefits of bioactive food components. Such nutritional guidance is recommended for reinforcing physical performance and bolstering the immune system, particularly during chronic stages of AIDS progression when the balance of antioxidants and minerals is disrupted [70,71]. Increased oxidative stress during these stages can exacerbate HIV-1 progression. A well-planned diet, rich in antioxidants and essential nutrients, can mitigate this oxidative stress, potentially reducing the stimulation of HIV-1 replication [72]. This underscores the importance of personalized nutritional counseling as a key component of comprehensive HIV-1 point of care. Following the World Health Organization (WHO) recommendations [73] ensures the intake of the Recommended Nutrition Intake (RNI) for each required micronutrient supplement [73] for PLWH.

This scientific review focuses on the potential role and advantages of nutritional supplements for people living with HIV-1. Among these micronutrients, creatine stands out for its potential benefits for PLWH exploring its connection with the required balanced nutrition during retroviral replication and virus-related diseases. The current clinical status of creatine’s utilization in this context will also be discussed in this review.

Creatine, an organic compound with the nominal formula CNCH_2_CO_2_H, occurs naturally in human muscle cells [74,75]. Numerous studies have demonstrated its consistent ability to enhance strength, fat free mass, and improve muscle morphology [76–79]. Another illustrative example of nutritional significance in PLWH involves examining the mechanisms of inflammation that contribute to cardiovascular and bone diseases within this population. Such correlations often relate to nutritional abnormalities. For instance, cardiovascular disease-related abnormalities in food intake include dyslipidemia, insulin resistance, and increased visceral adiposity [80–83]. Similarly, bone disease-related issues are associated with factors like calcium intake, vitamin D status, and gonadal function [84].

Ensuring a well-balanced diet is needed for preventing and managing both viral and bacterial infections. Probiotics, for instance, play a role in maintaining mucosal surface integrity, enhancing antibody responses, and increasing blood cell production [85,86]. Precise modulation of the inflammatory response is important for achieving an adequate antiretroviral immune response in clinical settings. Substantial efforts have been directed towards demonstrating that the antiretroviral properties of specific compounds, such as resveratrol, rapamycin, and metformin, through *in vitro* studies [87,88] (using mammalian cells) and *in vivo* experiments (utilizing humanized mouse models) [87], indicate a potential therapeutic role for these agents in HIV-1 management.

Physicians must place heightened emphasis on the potential cardiovascular risks associated with very low-carbohydrate (VLC) and ketogenic diets (KDs), particularly concerning the elevation of low-density lipoprotein (LDL) cholesterol levels. While Kirkpatrick *et al*. [89] have indicated that VLC/KDs can lead to more substantial short-term weight loss, especially within the first 6 months of dietary regimen, compared to higher carbohydrate diets, it is recommended to evaluate these benefits against the potential for increased cardiovascular risk over time. Over a 12-months of dietary regimen period, no significant difference in weight loss was observed between these diets. LC and KDs are widely recommended for controlled weight loss, lipid management, and in managing insulin resistance and type II diabetes [90,91]. These diets have shown improvements in triglyceride and high-density lipoprotein (HDL) cholesterol levels. However, a big concern arises with the observed variability in LDL cholesterol levels. In certain individuals, particularly those with latent genetic dyslipidemias, a higher increase in LDL cholesterol has been detected [92,93]. This elevation poses a significant risk for cardiovascular diseases, as elevated LDL cholesterol is a well-known risk factor for atherosclerosis and other cardiac conditions [94,95].

Recent advancements in understanding the impact of HIV-1/AIDS on chronic inflammation and metabolism have enhanced the management of chronic HIV-1 infection. Notably, well-nourished HIV-1 patients with a controlled viral load exhibit improved chances for management of the HIV-1 infection and delay its progression [96,97].

In the early years of the HIV-1 pandemic, prior cART options were not available, loss of lean tissue was progressing with the progression of the HIV-1 infection. Contrarily, with the implementation of the newest cART strategies, there is no or slight change in lean tissue mass [98]. Nutritional management and patients’ proper knowledge of the topic play an important role in enhancing the lifespan and the quality of life for PLWH.

## HIV-1 Infection and Nutrition

### HIV-1 infection and nutritional status

In chronic HIV-1 infection, a complex interplay between appetite reduction and malnutrition emerges on the physiological and psychological fronts, influencing both the progression of the disease and the patient’s quality of life. The infection itself often results in a decreased appetite, directly impacting adequate nutritional intake and heightening the risk of undernutrition. This decreased appetite significantly contributes to an increased vulnerability to malnutrition among HIV-1 patients. Concurrently, there’s also a risk of dietary mismanagement, where individuals may adopt imbalanced diets due to inadequate nutritional guidance or to counteract weight loss. Such dietary practices, while temporarily addressing certain nutritional deficits, can lead to a broader imbalance in essential nutrients, further exacerbating malnutrition risks. Therefore, addressing both the natural decline in appetite and the necessity for a nutritionally enriched while balanced diet is in effective nutritional management for HIV-1 patients [53,54]. Scientific consensus highlights the impact of dietary interventions on both innate and functional immune systems [92]. Malnutrition, extending beyond PLWH, correlates with compromised immune responses [99–101]. In the context of HIV-1, suboptimal nutritional status emerges as a robust predictor of survival rates, emphasizing the link between unintentional weight loss and heightened risk of mortality [10,102]. A deficient diet and nutritional insufficiency compromise the immune system, amplifying susceptibility to opportunistic infections [103,104].

Weight loss is a prevalent concern among HIV-1 patients and is indicative of an unfavorable disease prognosis [101]. The necessity of a wholesome diet, physical activity, and targeted education to mitigate nutritional deficits is underscored to foster skeletal muscle growth and overall well-being [10]. Particularly in presence of metabolic disorders, dyslipidemia, insulin-resistance, and conditions associated with increased cardiovascular risk during disease progression, a heightened focus on dietary consideration is warranted [81–83].

Kim *et al*. [100] found that within the HIV-1 population, subgroups with subpar dietary habits are more susceptible to unfavorable clinical outcomes. Their multivariate analysis indicated that the inability to meet the recommended dietary allowances (RDAs) is more of a socioeconomic rather than a clinical phenomenon, with reduced appetite being the sole clinical factor significantly associated with this challenge [100]. Individuals without caregivers, from minority backgrounds, and adults lacking support in purchasing groceries tended to demonstrate less satisfactory dietary intakes. [102]. Consequently, dietary education emerges as a crucial consideration for HIV-1 patients.

The phenomena of food insecurity and malnutrition emerge as significant barriers to the consistent adherence of cART in HIV-1 patients. Food insecurity, characterized by an unreliable access to adequate and nutritious food, can undermine the ability of patients to adhere to prescribed cART regimens. It is due to the absence of a stable nutritional foundation, which is essential for optimizing drug efficacy and mitigating adverse effects. Concurrently, malnutrition, stemming from inadequate dietary intake or poor nutrient absorption, further exacerbates this challenge by compromising the patient’s overall health status and potentially influencing drug metabolism and effectiveness. Together, these factors create a complex situation where the lack of nutritional stability can directly impact the efficacy of cART, thus hindering the therapeutic management of HIV-1 and posing a substantial obstacle to achieving optimal treatment outcomes [94–96]. Maintaining an appropriate balance of macronutrients and micronutrients through a healthy diet becomes essential for supporting the body’s immune functions during cART treatment. Additionally, certain antiretroviral drugs require administration with food. Non-compliance with nutritional recommendations among HIV-1 patients often correlates with an elevated risk of drug-related side effects, including appetite loss, with negative consequences for health, body composition, and muscle strength. Moreover, malnutrition adversely affects the metabolism and efficacy of antiretroviral medications [10].

In the context of managing HIV-1 infection, a diet should comprise substantial portions of vegetables, fruits, whole grains, and legumes, along with lean proteins, while being limited in added sugars and trans-fats. This approach aligns with general healthy dietary principles, which are essential for everyone, including individuals living with HIV-1. According to the WHO, a healthy diet helps protect against malnutrition in all its forms and noncommunicable diseases (NCDs), including diabetes, heart disease, stroke, and cancer [105]. It is recommended to limit the intake of free sugars to less than 10% of total energy intake, and ideally less than 5% for additional health benefits. The total energy intake from fats should be less than 30%, with a focus on unsaturated fats over saturated and trans-fats [105].

In PLWH, the strategic integration of Omega-3 essential fatty acids, particularly Eicosapentaenoic acid (EPA) and Docosahexaenoic acid (DHA), into their dietary regimen is of paramount importance [106–108]. These long-chain omega-3 polyunsaturated fatty acids (omega-3 LCPUFAs) are renowned for their anti-inflammatory properties. They are crucial in addressing the inflammatory processes inherent in HIV-1 pathology [109]. While direct evidence linking EPA and DHA to the specific attenuation of wasting syndrome in HIV-1 patients is still emerging, the general benefits of these fatty acids, including cardio- and neuroprotective effects, are well-documented in broader clinical nutrition and aging research [110,111]. These effects suggest a potential therapeutic role in mitigating the progression of HIV-1-related complications. However, it is important to acknowledge that the data concerning the impact of omega-3 on HIV-1 patients remain varied, influenced by factors such as dosage, treatment duration, baseline omega-3 status, and concurrent nutrient intake. Therefore, further well-designed clinical trials are necessary to elucidate the optimal application of EPA and DHA in HIV-1 management and to develop tailored nutritional strategies that enhance the overall health outcomes in this patient population [13].

### Wasting syndrome in HIV-1 patients

The wasting syndrome, also known as slim disease, first emerged in PLWH in Uganda in 1985 [112]. Defined by the Center for Disease Control and Prevention (CDC) as a >10% body weight loss over 6 months, with a 5% loss linked to decreased survival rates [113], this condition is strongly associated with lean body mass loss, elevating the risk of mortality and morbidity. In cART treated HIV-1 patients, a low BMI independently predicts increased mortality, despite improvements in immune function with cART treatments [114,115].

Weight loss is a prevalent issue in PLWH, with protein malnutrition and altered metabolism contributing to HIV-1-related weight loss and impairing T-lymphocyte function [116]. Elevated levels of pro-inflammatory cytokines, including tumor necrosis factor-alpha (TNF-α), interleukin-6 (IL-6), and interleukin-1 (IL-1), play a pivotal role in wasting syndrome [117,118]. Apart from suppressing appetite, these cytokines promote proteolysis, potentially contributing to muscle wasting through upregulation of the ubiquitin-proteosome pathway [119]. Increased plasma triglycerides may result from cytokine stimulating liver lipogenesis [116,120]. In addition, inhibition of lipoprotein lipase by high TNF-α circulating levels can occur, decreasing adipose tissue content which may also contribute to the wasting syndrome [121]. Furthermore, higher circulating proinflammatory cytokines in HIV-1 wasting syndrome individuals promote an increased resting energy expenditure (REE), also observed in the early stage of the infections [122].

Coodley *et al*. [98] demonstrated that wasting syndrome in HIV-1 patients is marked by diminished serum levels of folate, vitamin A, and carotene [98]. Supplementation with micronutrients, including vitamin B-12, has been shown to decelerate HIV-1 disease progression and enhance symptomfree survival [122]. Early stages of HIV-1infection often coincide with nutritional deficiencies, with reduced plasma levels of zinc, vitamins B-12, B-6, A, and E linked to impairments in immune response and cognitive functions among patients [92,99,123].

Several studies propose that nutritional status can exert influence across all phases of AIDS [10,92,99–102]. Malnutrition, intensified by inadequate food intake, impaired nutrient absorption, and altered metabolism, significantly hampers immune function, hastening the progression of HIV-1.

In a study conducted by Berneis *et al*. [124], researchers investigated the effects of nutritional supplements combined with dietary counseling in HIV-1 patients. Over a period of 12 weeks, the study demonstrated a significant anticatabolic effect of these interventions. This was evidenced by a reduction in whole-body protein catabolism, leading to positive changes in body composition, such as increased lean mass and decreased fat mass. These findings highlight the potential of nutritional interventions in enhancing the overall metabolic health and body composition of individuals living with HIV-1. In the comprehensive management of HIV-1 patients, it is recommended to implement systematic and detailed assessments encompassing biochemical nutritional status, anthropometric measurements, and body composition analysis. This multifaceted approach is fundamental to detect and address the nuanced metabolic alterations and nutritional deficiencies often associated with HIV-1 infection and antiretroviral therapy [125]. Regular monitoring of biochemical markers, including micronutrient levels, lipid profiles, and inflammatory markers, provides useful insights into the metabolic health of these patients. Additionally, anthropometric assessments, such as body mass index (BMI), waist-to-hip ratio, and skinfold thickness, offer valuable information on changes in body weight and fat distribution, which are pivotal for identifying risks related to wasting syndrome and lipodystrophy [125]. Furthermore, advanced body composition analysis techniques, like dual-energy X-ray absorptiometry (DEXA) or bioelectrical impedance analysis (BIA), allow for a more comprehensive evaluation of muscle mass, fat mass, and bone density, thus enabling targeted nutritional and therapeutic interventions [126,127]. These monitoring tools play an integral role in understanding and addressing wasting syndrome, offering a hope for improved outcomes in HIV-1 patient care.

### HIV-1 infection and zinc

Zinc (Zn) is an integral component to various immune processes, including T-cell division, maturation, differentiation, lymphocyte response to mitogens, programmed cell death, gene transcription, and membrane function [128]. Notably, zinc forms a structural component of numerous proteins, neuropeptides, hormone receptors, and polynucleotides, and is vital to produce certain hormones and enzymes like copper (Cu), Zn superoxide dismutase and thymulin, which are essential for T-lymphocyte formation [129,130]. Maintaining adequate zinc levels is important for optimal immune function, as zinc deficiency has been demonstrated to suppress both humoral and cell-mediated immune responses [131]. Such a deficiency, characterized by lymphopenia and thymic atrophy, has been associated with an increased frequency of infections [132,133]. In both animals and humans, zinc deficiency leads to rapid thymic atrophy, compromised cell-mediated cutaneous sensitivity, and lymphopenia, impacting primary and secondary antibody responses, especially for antigens requiring T-cell help. This deficiency also diminishes antibody responses and the generation of splenic cytotoxic T-cells post-immunization, while inhibiting the production of tumor necrosis factor implicated in the pathophysiology of cachexia and wasting in AIDS [134].

Recent research [123,135–138] indicates that zinc supplementation plays a role in slowing the course of HIV- 1 disease, reducing opportunistic infections both with and without antiretroviral therapy, and significantly lowering HIV-1-related mortality when combined with multivitamins and selenium [139]. It’s noteworthy that dosages exceeding the FDA (Food and Drug Administration) Daily Values may, however, exacerbate HIV-1 disease progression [136]. Several studies have investigated the relationship between HIV-1 infection and zinc levels, shedding light on the potential impact of zinc on the progression and severity of the disease.

Baum *et al*. [123] demonstrated through their study that long term zinc supplementation at nutritional levels can delay immunological failure and decrease diarrhea over time in HIV-1 positive adults. This evidence supports the prescription of zinc therapy as a safe, convenient, and cost-effective approach to enhance immune response. Baum’s study could further explore to potentially establish use of zinc as a promising candidate for exploration as an adjuvant therapy in the future [123]. A study by Jones *et al*. [140] found that zinc deficiency was associated with increased viral load and accelerated disease progression in PLWH. This observation is supported by a comprehensive review by Smith and Wesseling [141], which highlighted the important role of zinc in modulating immune function and its potential in managing HIV-1 infection. Moreover, a clinical trial conducted by Patel *et al*. [142] demonstrated that zinc supplementation has a potential role on viral replication and immune function in PLWH. These findings collectively suggest a nuanced correlation between zinc status and HIV-1 pathogenesis. While further research is needed to elucidate the underlying mechanisms, these studies underscore the importance of considering zinc supplementation as a recommended adjunct therapy in the management of HIV-1 infection.

### HIV-1 infection and vitamin D

Vitamin D is an essential component for bone health and has long been associated with difficulties in bone mineralization. Beyond its impact on skeletal health [143], Vitamin D influences a broad spectrum of extra-skeletal health outcomes, including muscle function, immune function, cardiovascular disease, diabetes, and cancer [144–146]. The global prevalence of vitamin D deficiency has sparked increased interest in its potential benefits through supplementation across various infectious and chronic disorders [147–149].

Multiple studies indicate that individuals with HIV-1, like the general population, often experience vitamin D insufficiency [150,151]. The clinical significance of vitamin D in HIV-1-infected patients has attracted significant attention due to parallels with the risk of chronic disease outcomes observed in vitamin D deficiency. This holds significant relevance, especially when considering its implications for the immunological function in the progression of HIV-1 and susceptibility to opportunistic infections [152,153].

Vitamin D’s immunomodulatory effects arise from its autocrine transit in human monocytes and macrophages, activating the toll-like receptors (TLRs1/2, TLR4) and interferon receptors (IFN- and CD40) [154]. Through up-regulating Vitamin D Receptor (VDR) and CYP27B1, these receptors initiate a signal cascade converting of 25(OH)D to 1,25(OH) D. During infection, monocyte and macrophage functions are regulated by 1,25(OH)D binding to the VDR, influencing multitarget gene expression. Vitamin D also hinders the development of dendritic cells (DCs), thereby reducing the inflammatory response to viral diseases [155].

Elevated levels of vitamin D and the vitamin D receptor (VDR) have been implicated in contributing to innate resistance against HIV-1 infection [156,157]. This association is thought to be mediated through several mechanisms, including the upregulation of interleukin-10 (IL-10). IL-10 is a critical cytokine in the immune system, known for its antiinflammatory properties and its role in immune regulation. Its increased presence could play a vital role in modulating the immune response against HIV-1 [158,159]. Additionally, there is evidence that suggests the upregulation of IL-10. It may be accompanied by the activation of anti-HIV-1 defensins in the mucosal tissues, which are among the first lines of defense against HIV-1 entry in individuals [160]. These defensins [161,162] are small cationic peptides that exhibit potent antiviral properties, and their activation in the mucosa represents a key component of the mucosal immune defense against HIV-1. Thus, the interplay between vitamin D, VDR, IL-10, and mucosal defensins forms a complex but potentially effective natural barrier against HIV-1, especially in individuals who are not otherwise protected against the virus. The VDR expression is closely associated with the upregulation of many anti-HIV-1 compounds, including cathelicidin microbial peptide (CAmP) and RNase 7, contributing to natural HIV-1 resistance [156,163]. Vitamin D in monocytes diminishes HIV-1 risk by impeding viral entry, restricting CD4 receptor expression, and reducing monocyte proliferation. TLR8 agonists are expected to inhibit HIV-1 infection via a CAMP and vitamin D-mediated autophagy pathway in macrophages [164,165]. Additionally, vitamin D induces autophagy in macrophages, acting as a barrier against HIV-1 infection [156]. Campbell and Spector’s research [166] has demonstrated that treatment with vitamin D, which is known to enhance autophagy, inhibits HIV-1 infection in human macrophages. This antiviral effect is further supported by studies indicating that autophagy-enhancing drugs, including vitamin D, reduce mucosal HIV-1 acquisition and suppress viral replication *ex vivo* [153,167,168]. In PLWH, vitamin D deficiency has been increasingly recognized as a critical factor exacerbating disease progression. This deficiency precipitates an over-secretion of a range of cytokines, notably CXCL10, IL-6, TNF-α, and D-dimer. Such an elevated cytokine milieu signifies a heightened inflammatory state, which is detrimental to the physiological integrity and immune competence of PLWH. Accompanying this cytokine surge is the activation of hyperactive monocyte subpopulations, specifically those expressing CCR2 and CX3CR1. These activated monocytes are implicated in causing extensive tissue damage, thus weakening the body’s defenses against opportunistic infections, and accelerating the transition to AIDS. The consequence of this cascade is profound, as it not only exacerbates the clinical progression of HIV, but also significantly increases the risk of mortality.

Considering clinical guidelines, the recommended daily intake of vitamin D is typically suggested to be between 1500 and 2000 International Units (IU) per day for the general population. However, for individuals who are HIV-1 positive, the advised dosage is substantially higher, ranging from 6,000 to 10,000 IU per day. This increased recommendation is based on the understanding that HIV-1 positive individuals have a greater need for vitamin D, both for its immunomodulatory effects and to counteract the accelerated bone density loss often associated with both the disease and antiretroviral therapy. Additionally, in the context of monitoring and treatment, a serum 25-hydroxyvitamin D3 (25(OH)D3) concentration below 20 ng/mL (50 nmol/L) is considered indicative of hypovitaminosis D [169].

In an insightful study conducted by Alvarez *et al*. [153] significant findings were made regarding the impact of normalizing vitamin D levels in PLWH. The study revealed that achieving optimal vitamin D levels in these patients, irrespective of their combination antiretroviral therapy status, resulted in a notable reduction of inflammatory markers that are typically associated with bone turnover. This is particularly crucial, as it suggests a decreased risk of developing secondary hyperparathyroidism, a condition often seen in HIV-1 patients that can lead to serious bone issues. Furthermore, the study highlighted another critical aspect of vitamin D normalization – the enhancement of the anti-bacterial response. This finding underscores the broader immunological benefits of vitamin D in HIV-1-infected patients, beyond its traditional role in bone health. These results not only provide a deeper understanding of the multifaceted role of vitamin D in HIV-1 management, but also emphasize the importance of maintaining adequate vitamin D levels. It is a part of a comprehensive approach to treating HIV-1-infected individuals, aiming to improve their overall health outcomes and reduce the risk of complications associated with the infection and its treatment.

### HIV-1 infection and vitamin B-12

Recently, vitamin B-12 has garnered attention from scientists and the public alike due to its intricate biological function and its role in modifying cellular immunity by enhancing CD8^+^ T and Natural Killer (NK) cells activity [170]. Deficiency in vitamin B-12 has been associated with decreased NK cell activity and a reduction in circulating lymphocytes [167]. Supplementation with this vitamin can alter these immunological responses, increasing CD8^+^ T cells and NK cells activity, as well as elevating white blood cell and lymphocyte counts in rats on a low protein diet [171,172].

Several studies have examined the relationship between vitamin B-12 levels and AIDS progression, shedding light on potential immunomodulatory effects. A study by Tang *et al*. [173] found that the low vitamin B-12 levels were associated with increased viral load and decreased CD4^+^ T cell counts in PLWH. This observation suggests the potential importance of maintaining optimal vitamin B-12 status in the context of HIV-1 infection.

Furthermore, a randomized controlled trial conducted by Baum *et al*. [174] investigated the impact of vitamin B-12 supplementation on immune parameters PLWH. The results suggested that supplementation potentially led to improvements in certain immunological markers, highlighting the role of vitamin B-12 as a supportive intervention in managing HIV-1 infection. However, it is essential to note that further well-designed clinical trials are warranted to explore and then establish the efficacy and safety of vitamin B-12 supplementation in this context. Todorova *et al*. [175] suggest that vitamin B-12 might serve as a viable immunotherapeutic tool, although it does not alter immunoglobulin levels [175].

The involvement of cobalamin, a form of vitamin B-12, in cancer immunotherapy has shown positive effects on anticancer defense [176,177]. However, the accumulated evidence indicating an increased cancer risk associated with high vitamin B-12 levels complicates its immunological role [178–180].

Studies reveal that 20% of AIDS patients have insufficient vitamin B-12 levels, leading to higher risk of hematologic and neurologic impairment [181]. The initiation of antiretroviral medication has been proven to influence patients’ vitamin B-12 levels. Most investigations on vitamin B-12 status focus on late-stage disease exacerbated by factors such as tuberculosis, neuropsychiatric symptoms, and others [182–187]. Given the overlapping of HIV-1 infection and vitamin B-12 insufficiency, early detection and treatments are needed to prevent permanent damage.

According to Kavitha *et al*. [176] findings, vitamin B-12 is a helpful modulator of the immunological and inflammatory condition of HIV-1-infected patients. It should be included as a part of a sensible and nutritious diet for HIV-1-infected individuals to ensure optimal immune system function and avoid potential detrimental consequences.

The connection between HIV-1 infection and vitamin B-12 levels is a complex and intriguing area of ongoing research that requires more evidence and data from clinical trials. While some studies point towards a potential benefit of maintaining adequate vitamin B-12 levels in PLWH, the current evidence is not conclusive and needs more evidence. Future research should aim to clarify the mechanisms underlying vitamin B-12 role in HIV infection and explore its full benefits for clinical management.

### HIV-1 infection and Mediterranean diet

The Mediterranean diet (MD), characterized by a high intake of legumes, whole grains, fruits, vegetables, nuts, extra virgin olive oil, and fish, and a low consumption of red meat, processed meats, and ultra-processed foods, has been extensively studied for its beneficial health impacts [188–192]. Notably, strong adherence to the MD is linked with reduced risks of CVD, type 2 diabetes mellitus (T2DM), and metabolic syndrome [193–195]. These benefits are primarily attributed to the diet’s anti-inflammatory and antioxidant properties, which are important to manage chronic diseases.

The HIV-1 infection and dietary strategies, particularly the Mediterranean diet, has emerged as a fascinating area of research within the realm of HIV-1 care and management. Numerous studies have investigated the potential impact of MD on the progression of HIV-1 and the overall well-being of people living with the virus. A notable study by Estruch *et al*. [196] conducted a randomized trial examining the effects of a MD supplemented with extra-virgin olive oil or nuts on the prevention of major cardiovascular events among individuals at high cardiovascular risk, a population that often intersects with those living with HIV-1. The findings not only highlighted the cardiovascular benefits of the Mediterranean diet but also hinted at potential implications for individuals with HIV-1. Ongoing exploration into the immunomodulatory aspects of the Mediterranean diet in the context of HIV-1 infection has been carried out by Jones *et al*. [197]. Their study revealed a positive association between adherence to the Mediterranean diet and higher CD4^+^ T cell counts among PLWH. This observation suggests a potential link between dietary habits and better immune system function, shedding light on the broader impact of lifestyle choices when it comes to managing HIV-1.

A study conducted by Pastor-Ibanez *et al*. [198] is particularly significant about MD role in HIV-1 infection. This research demonstrated that a MD regimen, supplemented with 50 g/day of extra virgin olive oil (EVOO) and 30 g/day of walnuts for over 12 weeks, yielded significant improvements in metabolic parameters, immune activation, T-Reg function, and gut microbiota composition in HIV-1-infected people [198]. The findings of this study are noteworthy as they contribute to a growing body of evidence suggesting the potential magnitude of dietary interventions in managing HIV-1 related complications. Of particular interest in Pastor-Ibanez *et al*. [198] study is the reported alteration in gut microbiota, specifically a significant reduction in Bacteroides levels among individuals with high adherence to the MD. This change positively influenced the Prevotella/Bacteroides ratio, a marker often associated with gut health and systemic inflammation. The gut microbiome plays a critical role in immune regulation and is particularly relevant in HIV-1 infection, where gut integrity and microbial balance are often compromised. The improvement in the Prevotella/Bacteroides ratio indicates a healthier gut environment, which could have far-reaching implications for the overall health status of HIV-1 infected patients. Additionally, a comprehensive review by Smith and Smith [199] delves into the broader implications of dietary options, including the MD, in chronic viral infections. While not exclusively focused on HIV-1, their narrative provides insights into the potential role of diet in influencing immune responses and viral replication. It emphasizes the need for more research to elucidate the specific mechanisms through which dietary factors may affect the progression of HIV-1 disease.

The relationship between HIV-1 infection and the Mediterranean diet is a multifaceted subject that encompasses cardiovascular health, immune function, and overall wellbeing. While intriguing findings suggest potential benefits, it is crucial to interpret these results with big caution, while recognizing the complexity of HIV-1 infection and HIV-1 latency. There is the urge for further research and more data to establish dietary recommendations for individuals living with HIV-1.

## Conclusions

In summary, this review highlights the important role of well-balanced nutrition in effectively managing HIV-1infection and its related complications. A proper diet is recommended not only to boost the effectiveness of cART, but also to mitigate metabolic issues like lipodystrophy and insulin resistance, ultimately improving the overall quality of life for PLWH. Emphasizing beneficial nutritional strategies, when including Omega-3 fatty acids and vitamins D and B-12, is paramount in preventing wasting syndrome and addressing metabolic disorders. Regular dietary education and assessments of nutritional status are essential components for optimal HIV-1 patients care. We present a concise roadmap for implementing dietary interventions in HIV-1 treatment:

- Incorporate Nutritional Assessments: regularly assess the nutritional status of individuals with HIV as part of their routine care to identify deficiencies early.
- Tailored Nutritional Plans: develop individualized nutritional plans that address specific deficiencies, such as Zinc, Vitamin D, and vitamin B-12, and consider adopting a Mediterranean diet for its comprehensive benefits.
- Educate on Nutritional Interventions: provide education to patients on the importance of nutrition in managing HIV-1, including guidance on supplement use and dietary patterns.
- Monitor and Adjust: continuously monitor the impact of dietary interventions on the patient’s health and adjust the nutritional plan as necessary to optimize outcomes.

Despite the progress made, several areas require further exploration to fully understand the optimal dietary strategies for people living with HIV-1. Future research should focus on long-term outcomes of specific nutritional interventions, the interplay between diet, HIV-1 progression, and antiretroviral therapy effectiveness, and personalized nutrition plans tailored to individual patient needs. Identifying these gaps underscores the importance of continued investigation to enhance the well-being and treatment outcomes for PLWH including the well-balanced nutrition diets.

## Figures and Tables

**Table 1.** Structured table summarizing the beneficial impacts of zinc, vitamin D, vitamin B-12, and the Mediterranean diet on PLWH.

Nutrient/Factor	Impact on HIV-1 Infection	Mechanism of Action	Evidence	Recommendations
**Zinc**	Slows HIV-1 disease progression, reduces opportunistic infections	Immune system modulation, structural component of enzymes and proteins	Supplementation combined with multivitamins and selenium lowers HIV-1-related mortality	Dosages exceeding FDA Daily Values may exacerbate disease progression
**Vitamin D**	Enhances immune response, potentially reduces HIV-1 replication	Immunomodulatory effects through activation of toll-like receptors and vitamin D receptors	Associated with innate resistance against HIV-1, influences cytokine production	6,000 to 10,000 IU per day recommended for HIV-1 positive individuals to counteract bone density loss
**Vitamin B-12**	Associated with lower viral load and higher CD4^+^ T cell counts, potential in improving immune parameters	Enhances CD8^+^ T and Natural Killer (NK) cell activity, involved in cellular immunity	Low levels associated with increased viral load and decreased CD4^+^ T cell counts, supplementation suggested	Inclusion in a nutritious diet recommended for optimal immune system function
**Mediterranean Diet**	Improves metabolic parameters, immune activation, and gut microbiota composition in HIV-1-infected people	Anti-inflammatory and antioxidant properties, beneficial for cardiovascular health and immune function	Supplementation with extra-virgin olive oil and walnuts showed significant improvements in HIV-1 patients	High adherence to the diet recommended for better immune system function and overall well-being in PLWH
